# Mathematics anxiety and self-efficacy among students in Hong Kong and Macao (China): socioeconomic disadvantage as a moderator of academic achievement

**DOI:** 10.3389/fpsyg.2026.1981122

**Published:** 2026-10-01

**Authors:** Ping Fu, Wenxin Deng

**Affiliations:** School of Educational Sciences, Guangdong Polytechnic Normal University, Guangzhou, China

**Keywords:** Hong Kong, Macao, mathematics achievement, mathematics anxiety, mathematics self-efficacy, PISA 2022, socioeconomic disadvantage

## Abstract

**Purpose:**

This study examines whether socioeconomic disadvantage alters the associations of mathematics anxiety and mathematics-specific self-efficacy with mathematics achievement among adolescents in Hong Kong (China) and Macao (China). The research problem is not whether anxiety, efficacy, and socioeconomic status are individually related to achievement; those relationships are already well established. The unresolved question is whether socioeconomic disadvantage functions as a boundary condition on the two psychological-achievement slopes when anxiety and self-efficacy are modeled jointly.

**Data and methods:**

This quantitative, observational, cross-sectional secondary analysis uses public PISA 2022 student data for Hong Kong (China) and Macao (China). The source data contained 10,291 relevant student records; after complete-case screening, 9,760 students from 209 schools were analyzed. Mathematics achievement was represented by PV1MATH-PV10MATH, mathematics anxiety by ANXMAT, mathematics self-efficacy by MATHEFF, and socioeconomic disadvantage by reverse weighted-standardized ESCS. Models were estimated separately across all 10 plausible values with final student weights, and sampling variance was estimated with the 80 official Fay-BRR replicate weights before plausible-value pooling.

**Findings:**

Anxiety was negatively associated with mathematics achievement (*B* = −9.03, SE = 1.53, *p* < 0.001), self-efficacy was positively associated with achievement (*B* = 34.82, SE = 1.62, *p* < 0.001), and greater socioeconomic disadvantage was associated with lower achievement (*B* = −14.88, SE = 1.69, *p* < 0.001). Neither the anxiety-by-disadvantage interaction (*B* = −1.77, SE = 1.52, *p* = 0.244) nor the self-efficacy-by-disadvantage interaction (*B* = −0.46, SE = 1.42, *p* = 0.744) was statistically significant. Equal-jurisdiction weighting, inverse-probability attrition weighting, bottom-SES-quartile models, and jurisdiction-specific models produced the same substantive moderation conclusion.

**Contribution and practical relevance:**

The findings show concurrent psychological and socioeconomic gradients while placing the moderation conclusion within the official PISA variance framework. Interventions should address emotional, motivational, and structural barriers in parallel rather than assuming that socioeconomic disadvantage necessarily magnifies the psychological gradients.

## 1 Introduction

Mathematics achievement is often discussed as though it were the direct expression of accumulated knowledge. That view is incomplete. At the point of assessment, students also bring expectations about their own capability, emotional responses to mathematics, memories of prior successes and failures, and unequal access to resources that support learning. These conditions become particularly consequential in adolescence, when mathematics becomes more abstract, formal evaluation carries greater consequences, and students begin to make educational choices that can narrow or widen later participation in science, technology, engineering, and mathematics. A useful account of mathematics achievement, therefore, needs to connect cognitive performance with the emotional, motivational, and socioeconomic conditions under which students learn and are assessed.

Mathematics anxiety is among the most consistently documented psychological correlates of mathematics achievement. Early syntheses established a negative relationship between anxiety toward mathematics and performance (Hembree, 1990; Ma, 1999). Later work clarified that mathematics anxiety is not merely a synonym for low ability. It encompasses worry, tension, avoidance, apprehension, and physiological arousal that can interfere with attention and working memory, while repeated difficulties and poor performance can also increase later anxiety (Carey et al., 2016; Dowker et al., 2016; Ramirez et al., 2018). More recent meta-analytic evidence strengthens this basic conclusion. Barroso et al. (2021), integrating 747 effect sizes, reported a small-to-moderate negative association between mathematics anxiety and mathematics achievement, while Caviola et al. (2022) documented robust relationships between forms of academic anxiety and performance in a very large synthesis. The magnitude is not fixed, however. Namkung et al. (2019) found that measurement characteristics, task difficulty, and assessment consequences moderated the anxiety-performance relationship, and Zhang et al. (2019) reported stronger negative relations in Asian and older school samples than in several comparison groups. The empirical question has therefore moved beyond whether anxiety matters to the conditions under which its association with achievement becomes stronger or weaker.

Self-efficacy provides a second, conceptually distinct route into the same achievement problem. In social cognitive theory, self-efficacy refers to judgments about whether one can organize and execute the actions needed to perform a particular task (Bandura, 1997). It should not be confused with general self-esteem or with a broad statement of academic confidence. Domain specificity is especially important in mathematics because confidence in solving mathematical tasks is closer to the behavioral demands of mathematics performance than a global self-evaluation. Foundational meta-analytic and mathematics-specific work has linked self-efficacy to academic outcomes and problem solving (Multon et al., 1991; Pajares and Miller, 1994), while Usher and Pajares (2008) showed how mastery experiences, social persuasion, vicarious experience, and affective states contribute to efficacy beliefs in school settings. Contemporary motivational work continues to place efficacy within a dynamic system in which beliefs influence persistence and goal pursuit while performance feedback also updates beliefs (Schunk and DiBenedetto, 2021).

This reciprocal architecture is important for interpretation. Honicke and Broadbent (2016) concluded that academic self-efficacy is positively related to performance but highlighted weaknesses in causal and longitudinal evidence. Talsma et al. (2018), pooling longitudinal cross-lagged studies, found reciprocal relationships and reported that prior performance can be at least as important for later efficacy as efficacy is for later performance. The implication for PISA is straightforward: a positive cross-sectional coefficient for mathematics self-efficacy is informative about the conditional association between perceived capability and concurrent achievement, but it is not a causal effect of raising self-efficacy by one unit. Situated expectancy-value theory similarly stresses that competence beliefs are embedded in tasks, contexts, identities, and perceived costs rather than operating as detached personality traits (Eccles and Wigfield, 2020).

A third body of research concerns socioeconomic inequality. Socioeconomic status is related to learning through several intertwined processes, including access to books and technology, stable study environments, tutoring and enrichment, parental educational resources, school choice, and the capacity to absorb setbacks without sacrificing learning time. Classic developmental and meta-analytic work documented robust socioeconomic gradients in academic outcomes (Bradley and Corwyn, 2002; Sirin, 2005), while Berkowitz et al. (2017) showed that socioeconomic background, inequality, school climate, and achievement are connected at multiple levels. This relationship is also well established in China. Liu et al. (2020), synthesizing 78 independent samples and 215,649 students in China, reported a moderate positive relationship between family socioeconomic status and academic achievement, although the magnitude differed by outcome domain and historical period. Evidence that motivational beliefs can influence poverty-related achievement differences further demonstrates why structural and psychological explanations should not be treated as competing accounts (Claro et al., 2016).

Despite the maturity of these three literatures, they are often connected only additively. Socioeconomic status is included as a background control, after which attention returns to the coefficients for anxiety or self-efficacy. That approach answers whether psychological-achievement associations remain after adjusting for average socioeconomic differences. It does not address whether the psychological slopes themselves vary across socioeconomic conditions. This distinction matters. Two students with comparable mathematics anxiety may have different opportunities to compensate for that anxiety. One may have access to tutoring, a quiet study space, rapid teacher feedback, and repeated chances to rebuild mastery. Another may face the same emotional burden with fewer external resources. Similarly, strong self-efficacy might be easier to translate into persistent, strategic work when instructional resources are readily available, or it might become especially valuable under disadvantage as a compensatory motivational resource. Both patterns are theoretically plausible.

The present study treats socioeconomic disadvantage as a possible boundary condition rather than a nuisance covariate. This framing is motivated by the moderation evidence in the mathematics-anxiety literature, by the domain-specific logic of self-efficacy, and by the persistent socioeconomic gradient in Chinese educational achievement. It also addresses a contextual gap. Lee (2009) demonstrated that mathematics self-concept, self-efficacy, and anxiety are empirically separable across PISA 2003 systems and noted the coexistence of strong mathematics performance with relatively unfavorable self-beliefs in some Asian contexts. Foley et al. (2017) similarly emphasized the global reach of the mathematics anxiety-performance link. Yet these earlier PISA-related discussions predate the conditions represented in PISA 2022 and do not directly test whether socioeconomic position changes both anxiety-achievement and efficacy-achievement slopes within contemporary Chinese jurisdictions.

The study also addresses a methodological gap. PISA mathematics proficiency is represented by plausible values, not by a single error-free individual test score. Analyses that use only one plausible value can make results depend on an arbitrary draw and understate uncertainty. In addition, students are clustered within schools and sampled with unequal probabilities. A defensible secondary analysis must therefore preserve student weights, account for school clustering, and repeat estimation across the full plausible-value set. Interaction coefficients also require careful interpretation. A statistically significant interaction alone does not indicate where conditional associations are strongest, so simple slopes at substantively meaningful moderator values are necessary (Hayes, 2022).

PISA 2022 is particularly suitable for this question because mathematics was the major assessment domain and the student questionnaire contains the focal psychological and socioeconomic indicators used here. The OECD analytical framework defines the assessment architecture and the role of plausible values, while the PISA 2022 reports document the broader distribution of achievement, equity, and learning conditions across participating systems (Organisation for Economic Co-operation and Development, 2023a; Organisation for Economic Co-operation and Development, 2023b; Organisation for Economic Co-operation and Development, 2023c; Organisation for Economic Co-operation and Development, 2024a). The design remains cross-sectional for the constructs considered in this paper. Anxiety, self-efficacy, socioeconomic position, and achievement are observed in the same assessment cycle, so the study estimates conditional associations rather than temporal or causal effects.

Geographic precision is important because PISA 2022 does not include the China B-S-J-Z sample that appeared in PISA 2018. The present analysis therefore concerns students in Hong Kong (China) and Macao (China), the Chinese jurisdictions represented in the 2022 public-use data used here. All empirical claims are limited to these two jurisdictions and should not be generalized to a nationally representative Chinese adolescent population. Consistent with the title and analytical model, academic self-efficacy refers throughout this study specifically to mathematics self-efficacy, and achievement refers specifically to mathematics achievement.

Against this background, the study has four objectives. First, it estimates the conditional association between mathematics anxiety and mathematics achievement while accounting for mathematics self-efficacy, socioeconomic disadvantage, gender, age, grade, and jurisdiction. Second, it estimates the corresponding association between mathematics self-efficacy and achievement in the same model. Third, it tests whether socioeconomic disadvantage moderates either psychological-achievement association. Fourth, it evaluates robustness using a policy-readable bottom-SES quartile definition of disadvantage and jurisdiction-specific models.

The contribution is deliberately bounded. The study does not claim novelty for the established main associations of mathematics anxiety, mathematics self-efficacy, or socioeconomic status with achievement. Instead, its theoretical contribution lies in examining mathematics anxiety and mathematics-specific academic self-efficacy together within a common boundary-condition model in which socioeconomic disadvantage may alter their associations with mathematics achievement. Its empirical contribution lies in testing that model in the 2022 mathematics cycle for Hong Kong and Macao. Its methodological contribution is the preservation of all 10 mathematics plausible values and the estimation of sampling uncertainty with the 80 official Fay-BRR replicate weights rather than relying on a single score or conventional ordinary least squares shortcut. Its practical contribution follows from the pattern of evidence: if socioeconomic disadvantage changes the psychological slopes, interventions may need to be differentially targeted; if it does not, psychological and structural barriers should be treated as distinct but simultaneous targets.

## 2 Theoretical framework

### 2.1 Mathematics anxiety as an achievement emotion

There is a coherent explanation in control-value theory of why mathematics anxiety should be linked to achievement. The theory suggests that achievement emotions are a function of student appraisals of their control over achievement activities and their value of their achievement outcomes (Pekrun, 2006). Control-value theory predicts particularly high anxiety when achievement outcomes are important but perceived control over those outcomes is uncertain or low (Pekrun, 2006). Academic emotions have previously been studied at a program level in relation to self-regulation, learning, and achievement (Pekrun et al., 2002), and the Achievement Emotions Questionnaire was developed to provide a structured measure of achievement-related emotions (Pekrun et al., 2011). The need for separate measurement of achievement emotions, rather than lumping them all into one achievement affect, has been reiterated in more recent short form measurement studies (Bieleke et al., 2021).

A mathematics classroom would be a likely context for the control-value mechanism, as in mathematics learning, errors are visible, publicly judged, and higher-order tasks require significant working memory. Mathematics anxiety may redirect attention toward threat and failure-related worry, reduce the willingness to attempt challenging items, and promote avoidance. These mechanisms can still interfere even if knowledge is available. However, poor performance can lead to later anxiety because it can result in repeated failure. Carey et al. (2016) described this reciprocal uncertainty as the “chicken and the egg problem.” Because the present study is cross-sectional, it estimates concurrent associations and does not identify their temporal direction. The cross-sectional coefficient represents an association between anxiety and simultaneous achievement while controlling for the other variables in the model.

There is evidence for a negative expectation, but no single universal coefficient. In recent decades, Hembree (1990) and Ma (1999) established the general negative association early on, and Dowker et al. (2016) and Ramirez et al. (2018) reviewed the mechanisms, development, and possibilities for intervention. Barroso et al. (2021) estimated the overall correlation to be close to −0.28, while Caviola et al. (2022) included mathematics-related anxiety in a synthesis of academic anxiety and performance. Task difficulty and measurement features have been shown to be significant by Namkung et al. (2019), and stronger negative relationships were found in Asian samples by Zhang et al. (2019). The theoretical expectation is therefore that greater mathematics anxiety will be associated with lower mathematics achievement, while the magnitude of that association may vary across students, tasks, and contexts.

One possible contextual condition is socioeconomic disadvantage. Access to compensatory support following a challenging learning experience may be constrained by resource limitations. Students might not receive the same level of tutoring, access to high-quality instructional materials, or protected time for studying. Such impacts may be more pronounced if alternative supports are not readily available. This logic predicts a moderation effect: as socioeconomic disadvantage increases, the negative anxiety–achievement association may become stronger, corresponding to a negative anxiety-by-disadvantage interaction. However, the opposite scenario is also possible. When material resources are plentiful, and parents have high expectations, a competitive school culture, and high perceived stakes in mathematics, mathematics anxiety may be particularly consequential. Wang et al. (2015) also demonstrated that the mathematics anxiety-performance relationship might be motivational, revealing that emotional costs do not stand alone in the role of motivation. Thus, the sign of the interaction is an empirical question (though a negative interaction is hypothesized).

The Hong Kong and Macao context also warrants a bounded interpretation of this mechanism. Both are high-performing East Asian education systems represented separately in PISA, and the present analysis does not assume that findings from Western samples or China transfer unchanged to either jurisdiction. The moderation test therefore asks whether socioeconomic disadvantage differentiates the concurrent anxiety-achievement gradient within these two PISA 2022 systems. Because the data are cross-sectional, any observed association remains compatible with reciprocal processes in which prior mathematics difficulty or poor performance contributes to later anxiety.

### 2.2 Mathematics self-efficacy and perceived capability

In social cognitive theory, self-efficacy refers to an individual’s judgment of their capability to organize and execute the actions required to perform a specific task (Bandura, 1997). In this study, academic self-efficacy is operationally and conceptually restricted to mathematics: it denotes students’ perceived capability to perform mathematics tasks rather than general academic confidence, self-concept, or self-esteem. This domain-specific definition corresponds to the PISA MATHEFF indicator used in the analysis. Accordingly, references to academic self-efficacy throughout the manuscript should be understood as mathematics-specific academic self-efficacy.

Social cognitive theory explains why efficacy beliefs should be related to achievement. Students who believe they can manage mathematical tasks may be more willing to initiate challenging problems, persist after errors, regulate effort, seek feedback, and interpret difficulty as manageable rather than as indicative of fixed inability. Multon et al. (1991) provided early meta-analytic support for the relationship between efficacy beliefs and academic outcomes, and Pajares and Miller (1994) demonstrated the importance of self-efficacy in mathematical problem-solving. Usher and Pajares (2008) reviewed the major sources from which school students develop efficacy beliefs, including mastery experiences, vicarious experiences, social persuasion, and interpretations of affective or physiological states. Schunk and DiBenedetto (2021) place self-efficacy within a contemporary motivational system in which beliefs shape goal-directed action while experience continually recalibrates those beliefs.

The reciprocal nature of that system prevents simple causal interpretation. Honicke and Broadbent (2016) concluded that self-efficacy is positively associated with academic performance but stressed limitations in the evidence for directionality. Talsma et al. (2018) explicitly modeled both directions and found support for reciprocal relationships, with prior performance often making a substantial contribution to later efficacy. The same caution applies to mathematics. A high MATHEFF score may reflect genuine confidence that facilitates persistence, but it may also partly reflect accumulated success in mathematics. Cross-sectional PISA data cannot separate those processes.

Efficacy–anxiety is also important. Ahmed et al. (2012) reported a reciprocal relationship between mathematics self-concept and mathematics anxiety. Although self-concept is distinct from self-efficacy, this finding illustrates the reciprocal connections that can develop between mathematics-related beliefs and emotions. Lee (2009) showed that self-concept, self-efficacy, and mathematics anxiety were empirically separable from the data collected from PISA and could not be reduced to a single positive–negative attitude toward mathematics. This enables the modeling of ANXMAT and MATHEFF simultaneously. In the current model, the self-efficacy coefficient therefore represents its conditional association with mathematics achievement after adjustment for mathematics anxiety, socioeconomic disadvantage, and the specified controls.

The relationship between self-efficacy and achievement could also vary in relation to socioeconomic disadvantage. A resource-translation account proposes that confident students still need material and instructional resources to translate persistence and strategy use into successful learning; limited learning materials, instructional support, enrichment, or protected study time could therefore weaken the achievement return associated with self-efficacy. A compensatory-resource account predicts the opposite pattern, with self-efficacy becoming especially important under greater disadvantage. Local evidence also cautions against assuming that motivational processes operate identically across settings. Using PISA 2018 data from Macao, Ho (2025) reported that self-efficacy helped explain associations between wellbeing and academic achievement, while recent work with Hong Kong adolescents has likewise linked mathematics self-efficacy and anxiety to mathematics achievement (Zou, 2025). These findings support testing the proposed boundary condition directly within Hong Kong and Macao rather than importing a moderation pattern from Western samples. H5 retains the directional prediction that disadvantage weakens the positive self-efficacy-achievement association.

### 2.3 Socioeconomic disadvantage as a boundary condition

The substantive dimension of SES is typically represented as a background characteristic. Bradley and Corwyn (2002) explained the strong connection between socioeconomic status and developmental environments, and Sirin (2005) highlighted this very strong connection between socioeconomic status and academic achievement. Berkowitz et al. (2017) demonstrated that the socioeconomic context and inequality are related to school climate and achievement, and that the resource environment surrounding a student is not just a family characteristic. Liu et al. found that there is a long history of such a socioeconomic gradient in achievement in China, but that the gradient has shifted over time. The OECD’s PISA 2022 reporting also emphasizes equity, highlighting that it is important to include socioeconomic position as a substantive factor rather than as a nuisance adjustment (Organisation for Economic Co-operation and Development, 2023a).

The PISA index of economic, social, and cultural status (ESCS) can be helpful because it represents a broad socioeconomic context, which is not just one of its proxies. ESCS is standardized in the primary analysis using the final student weights and is sign-reversed (with higher scores representing higher disadvantage). This coding is directly interpretable in the direction of socioeconomic disadvantage by the moderator. Continuous specification is preferred as it retains information and avoids arbitrary thresholds. Given the nature of the data, a bottom-weighted quartile for each jurisdiction is simply used for sensitivity purposes as a comparison between the students in the lowest quartile of the local socioeconomic distribution and the three other quartiles that are relevant for policy.

The question of whether socioeconomic disadvantage operates as a moderator is distinct from the question of whether it has an independent association with achievement. The main effect compares the scores of students with lower SES resources to the scores of students with higher SES resources at the weighted means of the factors in the focal psychological predictors. The interaction terms determine whether the relationship between anxiety or efficacy and achievement varies across changes in socioeconomic disadvantage. Theoretically, these questions are separable. There is no interaction when a strong socioeconomic main effect is found, but the distribution of achievement is shifted lower without affecting the psychological gradients. On the other hand, if the main effect is weak and the interaction effect is strong, then the interaction may be the primary finding in the study while the main effect is secondary, since the consequences of anxiety and efficacy may vary significantly by socioeconomic position. One of the primary analytical contributions of the paper is distinguishing those patterns.

### 2.4 Integrated framework and hypotheses

Figure 1 summarizes the integrated model. Mathematics anxiety and mathematics self-efficacy are treated as distinct psychological correlates of achievement. Socioeconomic disadvantage has its own expected association with mathematics achievement and is simultaneously tested as a moderator of both psychological slopes. Gender, age, grade, and jurisdiction are included as limited controls, not as a claim that all potential confounders have been removed. The arrows in the framework represent theoretical and regression relationships rather than causal pathways.

**Figure 1:**
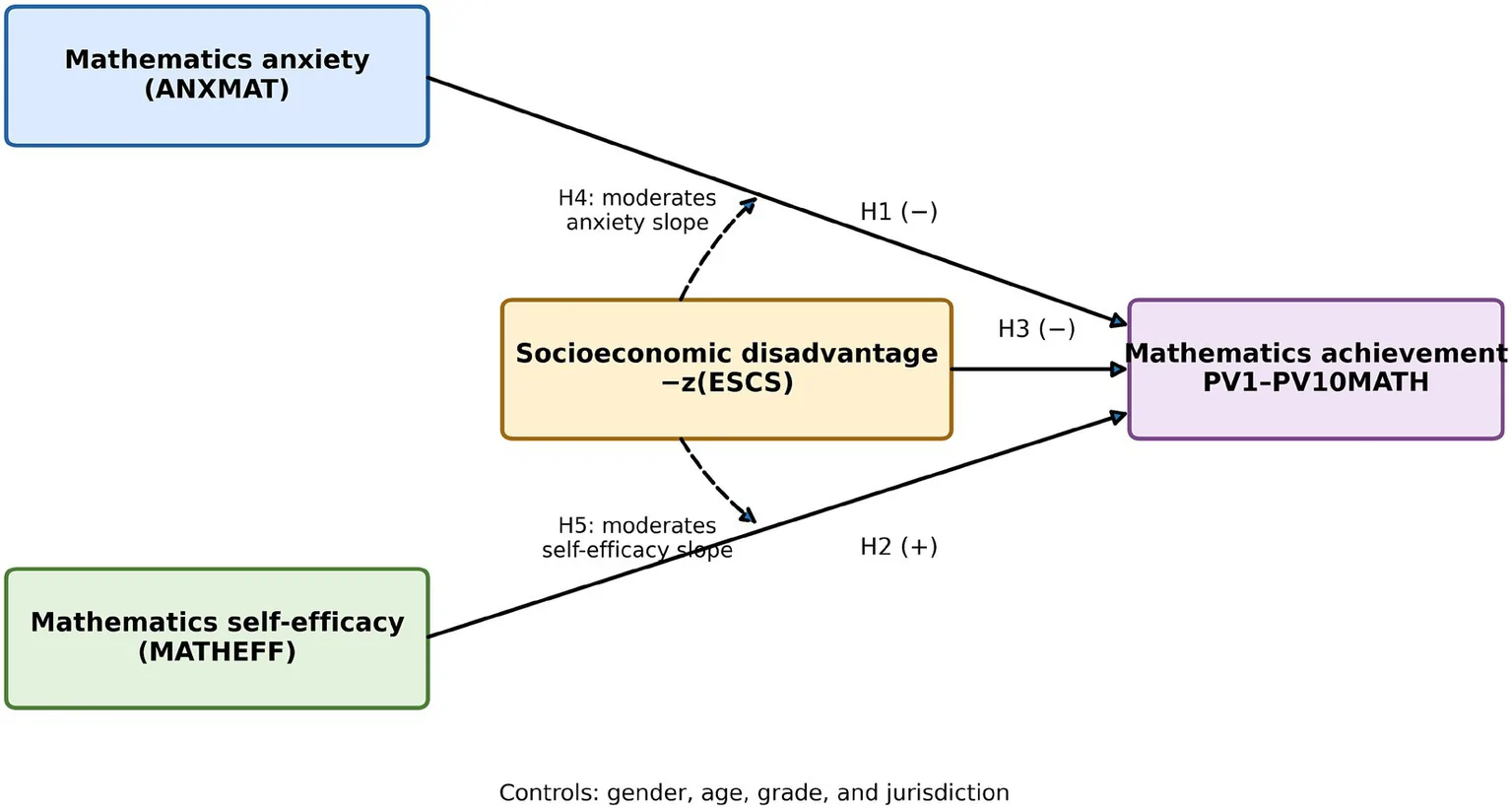
Conceptual model linking mathematics anxiety and mathematics self-efficacy to mathematics achievement, with socioeconomic disadvantage as the proposed moderator. Arrows denote theoretical/regression relationships, not causal effects.

Five hypotheses were formulated from the theoretical framework. H1 predicts that greater mathematics anxiety is associated with lower mathematics achievement. H2 predicts that stronger mathematics self-efficacy is associated with higher mathematics achievement. H3 predicts that greater socioeconomic disadvantage is associated with lower mathematics achievement. H4 predicts that socioeconomic disadvantage strengthens the negative association between mathematics anxiety and achievement, corresponding to a negative anxiety-by-disadvantage interaction. H5 predicts that socioeconomic disadvantage weakens the positive association between mathematics self-efficacy and achievement, corresponding to a negative self-efficacy-by-disadvantage interaction. Because the competing compensatory-resource account permits a positive self-efficacy-by-disadvantage interaction, the direction and confidence interval of this interaction are interpreted explicitly.

The integrated conceptual framework is presented in Figure 1.

## 3 Methods

### 3.1 Research design and data source

The study uses a quantitative, observational, cross-sectional secondary-data design based on the 2022 cycle of the Program for International Student Assessment (PISA). It examines student-level associations among mathematics anxiety, mathematics-specific self-efficacy, socioeconomic disadvantage, and mathematics achievement in Hong Kong (China) and Macao (China), with socioeconomic disadvantage specified as a moderator of the two psychological-achievement associations. The design is associational rather than experimental or longitudinal; all focal constructs were observed within the same assessment cycle, so the analyses estimate concurrent conditional associations and do not identify temporal ordering or causal effects. PISA is coordinated by the OECD and uses a two-stage probability sampling design in which schools are sampled first and eligible 15-year-old students are then sampled within schools. The PISA 2022 Assessment and Analytical Framework describes the mathematics assessment, questionnaire architecture, sampling logic, and use of plausible values for population-level proficiency inference (Organisation for Economic Co-operation and Development, 2023c). Mathematics was the major assessment domain in 2022, making this cycle appropriate for the present moderation analysis of mathematics-related psychological constructs and achievement.

The analysis draws on the public PISA 2022 student database for Hong Kong (China) and Macao (China). The source data provided 10,291 relevant student records and included the focal questionnaire indices, student and school identifiers, the final student weight, selected controls, and all 10 mathematics plausible values (PV1MATH-PV10MATH). The unit of analysis is the individual PISA student, and the relevant time period is the 2022 assessment cycle.

Before estimation, the data were checked for required variables, missingness, jurisdiction composition, duplicate student identifiers, and weight validity. All 10 mathematics plausible values and the final student weight were present; student identifiers were unique and all final weights were positive. Missingness was 4.43% for mathematics anxiety, 4.30% for mathematics self-efficacy, and 3.14% for ESCS; mathematics plausible values and the final student weight had no missing values. Complete-case restriction across the focal variables yielded 9,760 students in 209 schools (Hong Kong = 5,454; Macao = 4,306).

### 3.2 Measures

Mathematics achievement is the dependent variable and is represented by PV1MATH through PV10MATH. PISA plausible values are drawn from a posterior proficiency distribution intended for population inference; they should not be treated as 10 independent observed test scores for each student. The inferential model was therefore estimated separately for each plausible value. For all achievement statistics, each of PV1MATH-PV10MATH was analyzed separately, and the resulting statistic was pooled across plausible values. Plausible values were not averaged at the student level before computing means, standard deviations, correlations, regression coefficients, or standardized effect sizes. Figures use pooled model predictions rather than an individual-level mean-PV outcome.

Mathematics anxiety is measured with the PISA mathematics-anxiety index ANXMAT, derived from the ST292 questionnaire battery. The item content covers worry about mathematics difficulty, tension during homework, nervousness and helplessness while solving problems, concern about marks, and anxiety about failure. The study uses the OECD-scaled index rather than constructing an *ad hoc* mean from individual items. In the analytic sample, ANXMAT was standardized using the final student weights so that a one-unit difference in the regression corresponds to one weighted standard deviation of mathematics anxiety.

Mathematics self-efficacy is measured with MATHEFF, the PISA mathematics-specific self-efficacy index linked to the ST290/ST291 confidence batteries. These batteries ask students about their confidence in performing applied, formal, graphical, modeling, and interpretive mathematics tasks. MATHEFF therefore operationalizes academic self-efficacy as domain-specific mathematics self-efficacy. It was standardized with the final student weights in the same way as ANXMAT.

Socioeconomic disadvantage is derived from ESCS, the PISA index of economic, social, and cultural status. The primary moderator is the weighted-standardized ESCS score multiplied by −1. Higher values therefore indicate greater socioeconomic disadvantage. This sign reversal preserves the substantive information in ESCS while making the direction of the moderation terms consistent with the wording of the hypotheses. For robustness, a binary disadvantage indicator was created by identifying the bottom weighted ESCS quartile separately within Hong Kong and Macao. The weighted cut-points were −1.1859 in Hong Kong and −1.0986 in Macao, producing an overall weighted disadvantaged proportion of approximately 25%.

Controls were restricted to variables available consistently in the public-use data: gender, age, grade, and jurisdiction. Gender was based on PISA item ST004D01T, coded 1 = female and 2 = male. Male students were represented by an indicator variable, with female students as the reference category; age and grade were centered at their weighted analytic means, and Macao was coded as an indicator relative to Hong Kong. These controls reduce obvious compositional differences but do not provide causal identification.

### 3.3 Weighting, missing data, and plausible-value pooling

Final student weights were used for point estimates, with the 80 Fay-BRR replicate weights used for design-based variance estimation. The common analytic sample contained 9,760 of 10,291 relevant records, corresponding to 531 exclusions (5.16%). Missingness was 4.43% for ANXMAT, 4.30% for MATHEFF, and 3.14% for ESCS; gender, age, and grade had 0% missingness in the source records used for screening. Complete-case analysis was not justified solely by the low item-level percentages. Retained-versus-excluded students were compared on observed characteristics, and an inverse-probability-of-complete-case weighting sensitivity analysis was conducted to assess whether observable selection altered the focal moderation conclusions. At the survey level, Hong Kong’s PISA 2022 student response rate was 75%, while school response rates were 60% before replacement and 80% after replacement. These survey-level unit-response limitations are distinct from the additional 5.16% complete-case attrition in the present analysis and leave some potential for residual non-response bias.

Sampling variance was estimated with the 80 official PISA Fay-BRR replicate weights (W_FSTURWT1-W_FSTURWT80), while W_FSTUWT was used for full-sample point estimates. For each plausible value, the regression was estimated once with the final student weight and then 80 times with the replicate weights. Following the PISA 2022 Technical Report (Organisation for Economic Co-operation and Development, 2024b), the replicate-weight covariance was calculated as 0.05 times the sum of the outer products of the differences between each replicate estimate and the full-sample estimate. This design-based procedure reflects PISA’s stratification, clustering, unequal selection probabilities, and weight adjustments more directly than a school-clustered sandwich estimator.

For each of the 10 mathematics plausible values, the same weighted least-squares model was estimated using the final and replicate weights. Let q_m denote the full-sample coefficient vector for plausible value m and U_m its Fay-BRR covariance matrix.

The pooled coefficient is q-bar = (1/M) sum(m = 1 to M) q_m, with M = 10.

The within-plausible-value covariance is U-bar = (1/M) sum U_m. Between-plausible-value variability is B = Var(q_m).

Total covariance was calculated as T = U-bar + (1 + 1/M)B.

Standard errors are the square roots of the diagonal of T, so the reported uncertainty incorporates both design-based Fay-BRR sampling variance and variability across the 10 plausible-value estimates. Two-sided *p*-values were calculated from the pooled estimate and pooled standard error.

### 3.4 Model specification

The primary model for student i and mathematics plausible value m is:

$$\begin{array}{l} \text{MATH}\_\mathrm{im}=\mathrm{beta0}+\mathrm{beta1}\;\mathrm{ANX}\_i+\mathrm{beta2}\;\mathrm{EFF}\_i\\ +\mathrm{beta3}\;\mathrm{DIS}\_i+\mathrm{beta4}(\mathrm{ANX}\_i\;x\;\mathrm{DIS}\_i)+\mathrm{beta5}\\ (\mathrm{EFF}\_i\;x\;\mathrm{DIS}\_i)+{\text{gamma}}^{’}C\_i+\text{delta}\;\mathrm{MAC}\_i+\text{epsilon}\_\mathrm{im} \end{array}$$

where ANX is weighted-standardized mathematics anxiety, EFF is weighted-standardized mathematics self-efficacy, DIS is reverse weighted-standardized ESCS, C contains gender, centered age, and centered grade, and MAC distinguishes Macao from Hong Kong. Because all focal variables are centered, beta1 and beta2 represent the anxiety and efficacy slopes at average socioeconomic disadvantage, while beta3 represents the disadvantage association when anxiety and efficacy are at their weighted means. Beta4 and beta5 test whether the psychological slopes change with socioeconomic disadvantage.

The conditional anxiety slope at a disadvantage value d is beta1 + beta4 d, and the conditional self-efficacy slope is beta2 + beta5 d. Simple slopes were calculated at *d* = −1, 0, and +1, corresponding to relatively low disadvantage, mean disadvantage, and relatively high disadvantage. Their standard errors were calculated from the full pooled coefficient covariance matrix rather than by treating the main and interaction terms as independent.

Four sensitivity analyses were conducted. First, continuous disadvantage was replaced with bottom-ESCS-quartile membership defined within each jurisdiction. Second, the continuous moderation model was estimated separately for Hong Kong and Macao. Third, because final student weights make larger target populations contribute more heavily to a pooled estimate, all full and replicate weights were rescaled within each jurisdiction to equal totals, creating an equal-jurisdiction sensitivity analysis. Fourth, because complete-case exclusion was related to observable student characteristics, an inverse-probability-of-complete-case weighting analysis was conducted. Retention probabilities were modeled from mathematics achievement, gender, age, grade, and jurisdiction, and the resulting stabilized inverse-probability factor was multiplied by the final and replicate weights before re-estimating the 10 plausible-value models. The zero-order correlation matrix is reported only as descriptive bivariate context. Because complete-case status was defined by missingness in ANXMAT, MATHEFF, and ESCS, the unobserved values of the focal variables could not be entered directly into the complete-case retention model. The IPCW sensitivity analysis therefore addresses selection associated with observed achievement, demographic characteristics, and jurisdiction, but it cannot rule out missing-not-at-random selection related to the unobserved values of mathematics anxiety, mathematics self-efficacy, or socioeconomic status. This residual source of selection uncertainty is retained as a limitation of the analysis.

### 3.5 Ethical and reproducibility considerations

The study uses public, anonymized secondary data and involves no new recruitment, intervention, contact, or collection of identifiable personal information. Ethical approval was therefore not required for this secondary analysis. The dataset is publicly available from the OECD PISA 2022 database. The analysis script implements variable preparation, weighted standardization, all 10 mathematics plausible values, the 80 Fay-BRR replicate weights, equal-jurisdiction weighting, inverse-probability attrition weighting, sensitivity analyses, and figure generation.

## 4 Results

### 4.1 Sample characteristics and descriptive patterns

The final analytic sample contained 9,760 students from 209 schools, with 5,454 students from Hong Kong and 4,306 from Macao. The weighted mean age was 15.81 years, the weighted mean grade was 9.59, and 52.13% of the weighted sample were male. Mathematics descriptives were calculated separately for PV1MATH-PV10MATH and then pooled. The pooled weighted mathematics mean was 544.29 points, and the pooled within-plausible-value standard deviation was 102.67 points. The pooled jurisdiction means were 543.49 points in Hong Kong and 552.46 points in Macao. These jurisdiction means are unadjusted and should not be confused with the jurisdiction coefficient from the multivariable model.

Table 1 reports the descriptive statistics for the focal variables. Mathematics anxiety had a weighted mean of 0.239 (SD = 1.151), mathematics self-efficacy a weighted mean of −0.424 (SD = 1.255), and ESCS a weighted mean of −0.459 (SD = 0.998). The negative mean for MATHEFF is not interpreted as a deficit relative to an absolute scale origin; it reflects the OECD-scaled metric and the composition of the two-jurisdiction sample.

**Table 1:** Weighted descriptive statistics for the analytic sample (*N* = 9,760).

Variable	Weighted M	Weighted SD	Minimum	Maximum
Mathematics achievement	544.29	102.67	248.09	843.29
Mathematics anxiety (ANXMAT)	0.239	1.151	−2.395	2.635
Mathematics self-efficacy (MATHEFF)	−0.424	1.255	−3.508	2.356
ESCS	−0.459	0.998	−6.015	4.169
Age	15.81	0.287	15.25	16.33
Grade	9.59	0.616	7	12

Table 2 reports weighted zero-order correlations. Correlations involving mathematics achievement were calculated separately for each plausible value and then pooled rather than being computed from a student-level average of the 10 plausible values. Mathematics achievement correlated negatively with anxiety (*r* = −0.262), positively with self-efficacy (*r* = 0.421), and positively with ESCS (*r* = 0.239). Anxiety and self-efficacy were moderately negatively correlated (*r* = −0.472). ESCS correlated weakly with anxiety (*r* = −0.073) and modestly with self-efficacy (*r* = 0.172).

**Table 2:** Weighted zero-order correlations among focal variables.

Variable	1	2	3	4
1. Mathematics achievement	1.000			
2. Mathematics anxiety	−0.262	1.000		
3. Mathematics self-efficacy	0.421	−0.472	1.000	
4. ESCS	0.239	−0.073	0.172	1.000

### 4.2 Primary weighted moderation model

Table 3 presents the Fay-BRR and plausible-value-pooled weighted regression results. The model explained approximately 25.1% of the weighted variation in each plausible-value regression on average (mean R-squared = 0.251). At mean socioeconomic disadvantage, a one-weighted-standard-deviation increase in mathematics anxiety was associated with 9.03 fewer mathematics points, *B* = −9.03, SE = 1.53, 95% CI (−12.02, −6.04), *p* < 0.001. Relative to the PV-aware pooled mathematics standard deviation, this is approximately −0.09 SD. H1 was supported.

**Table 3:** Primary weighted moderation model pooled across 10 mathematics plausible values.

Term	*B*	SE	95% CI	*p*
Intercept	544.06	2.71	[538.75, 549.36]	<0.001
Mathematics anxiety (z)	−9.03	1.53	[−12.02, −6.04]	<0.001
Mathematics self-efficacy (z)	34.82	1.62	[31.63, 38.00]	<0.001
Socioeconomic disadvantage (z)	−14.88	1.69	[−18.19, −11.56]	<0.001
Anxiety × disadvantage	−1.77	1.52	[−4.74, 1.20]	0.244
Self-efficacy × disadvantage	−0.46	1.42	[−3.24, 2.32]	0.744
Male (female = reference)	0.77	2.94	[−4.99, 6.54]	0.792
Age (centered)	−15.11	6.09	[−27.04, −3.18]	0.013
Grade (centered)	35.69	2.83	[30.14, 41.24]	<0.001
Macao indicator	−1.40	2.74	[−6.77, 3.96]	0.608

Mean R-squared across the 10 plausible-value regressions = 0.251. Point estimates use W_FSTUWT. Sampling variance is estimated with W_FSTURWT1-W_FSTURWT80 using the Fay-BRR factor of 0.05 and then combined with between-plausible-value variability.

Mathematics self-efficacy showed the largest focal coefficient. A one-weighted-standard-deviation increase in MATHEFF was associated with 34.82 additional mathematics points, *B* = 34.82, SE = 1.62, 95% CI (31.63, 38.00), *p* < 0.001, corresponding to approximately 0.34 of the PV-aware pooled mathematics standard deviation. H2 was supported, although the cross-sectional design does not show that raising self-efficacy would itself produce a 34.82-point gain.

Socioeconomic disadvantage also had an independent negative association with mathematics achievement. A one-weighted-standard-deviation increase in disadvantage was associated with 14.88 fewer mathematics points, *B* = −14.88, SE = 1.69, 95% CI (−18.19, −11.56), *p* < 0.001, approximately −0.14 of the PV-aware pooled mathematics standard deviation. H3 was therefore supported.

The central moderation hypotheses were not supported. The anxiety-by-disadvantage interaction was negative in the hypothesized direction but statistically uncertain, *B* = −1.77, SE = 1.52, 95% CI (−4.74, 1.20), *p* = 0.244. H4 was therefore not supported. The self-efficacy-by-disadvantage interaction was *B* = −0.46, SE = 1.42, 95% CI (−3.24, 2.32), *p* = 0.744, indicating that H5 was not supported. As an explicit equivalence sensitivity check, a transparent practical margin of ±5 PISA points was used for a one-SD-by-one-SD interaction. With the pooled plausible-value-aware mathematics SD of 102.67 points, this margin corresponds to approximately 0.05 SD. The margin is treated as a deliberately small sensitivity threshold rather than as a universally established minimum important difference; its purpose is to distinguish interactions that are small on the PISA scale from effects large enough to alter the substantive interpretation of the focal gradients. The 90% CIs were (−4.26, 0.73) for anxiety by disadvantage and (−2.80, 1.87) for self-efficacy by disadvantage; both fell within the equivalence bounds (TOST *p* = 0.017 and *p* < 0.001, respectively). The corresponding 80% detectable interaction magnitudes were about 4.25 and 3.98 points, respectively. Thus, under this explicitly stated sensitivity margin, the data are inconsistent with interaction magnitudes of five points or larger while still allowing smaller interaction effects.

The control coefficients were secondary to the focal hypotheses. Male (female = reference) was not associated with a meaningful adjusted difference, *B* = 0.77, SE = 2.94, *p* = 0.792. Age was negatively associated with mathematics achievement conditional on grade and the other variables, *B* = −15.11, SE = 6.09, *p* = 0.013, whereas grade was positively associated with achievement, *B* = 35.69, SE = 2.83, *p* < 0.001. The adjusted Macao-Hong Kong indicator was small and statistically uncertain, *B* = −1.40, SE = 2.74, *p* = 0.608.

### 4.3 Simple slopes and interaction figures

The interaction coefficients indicate no statistically supported slope difference across disadvantage. For mathematics anxiety, the fitted association was −7.26 points at low disadvantage (−1 SD), SE = 2.33, 95% CI (−11.82, −2.70), *p* = 0.002; −9.03 points at mean disadvantage, SE = 1.53, 95% CI (−12.02, −6.04), *p* < 0.001; and −10.80 points at high disadvantage (+1 SD), SE = 1.96, 95% CI (−14.64, −6.96), *p* < 0.001. The numerical pattern becomes more negative with increasing disadvantage, but the Fay-BRR interaction test does not support a reliable difference among these slopes.

Figure 2 visualizes this result. The three adjusted lines are all downward sloping. Their modest divergence is consistent with the negative interaction estimate; however, the lines remain close enough that the pooled interaction confidence interval crosses zero. The appropriate conclusion is therefore not that disadvantage has no relevance. Disadvantage shifts predicted achievement downward through its strong main effect. What is unsupported is the narrower claim that it substantially changes the anxiety-achievement gradient.

**Figure 2:**
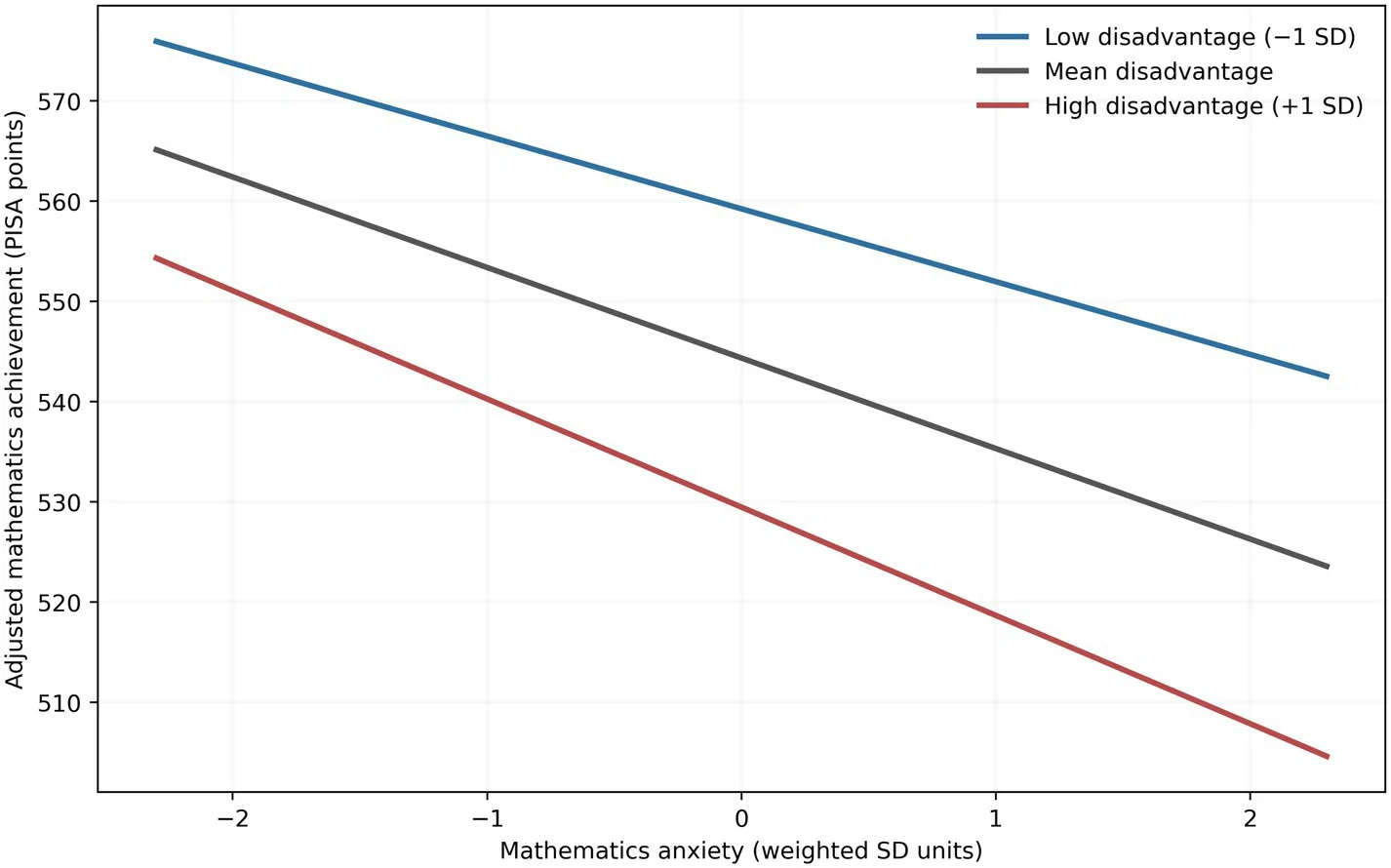
Adjusted mathematics achievement across mathematics anxiety at low, mean, and high socioeconomic disadvantage. The anxiety-by-disadvantage interaction was not statistically significant (*p* = 0.244).

The self-efficacy slopes were similarly stable. At low disadvantage, a one-SD increase in self-efficacy was associated with 35.28 additional points, SE = 2.47, 95% CI (30.43, 40.13), *p* < 0.001; at mean disadvantage, the slope was 34.82 points, SE = 1.62, 95% CI (31.63, 38.00), *p* < 0.001; and at high disadvantage, it was 34.36 points, SE = 1.79, 95% CI (30.86, 37.85), *p* < 0.001. Figure 3 therefore shows the fitted positive association without implying statistically different slopes across socioeconomic disadvantage (Table 4).

**Figure 3:**
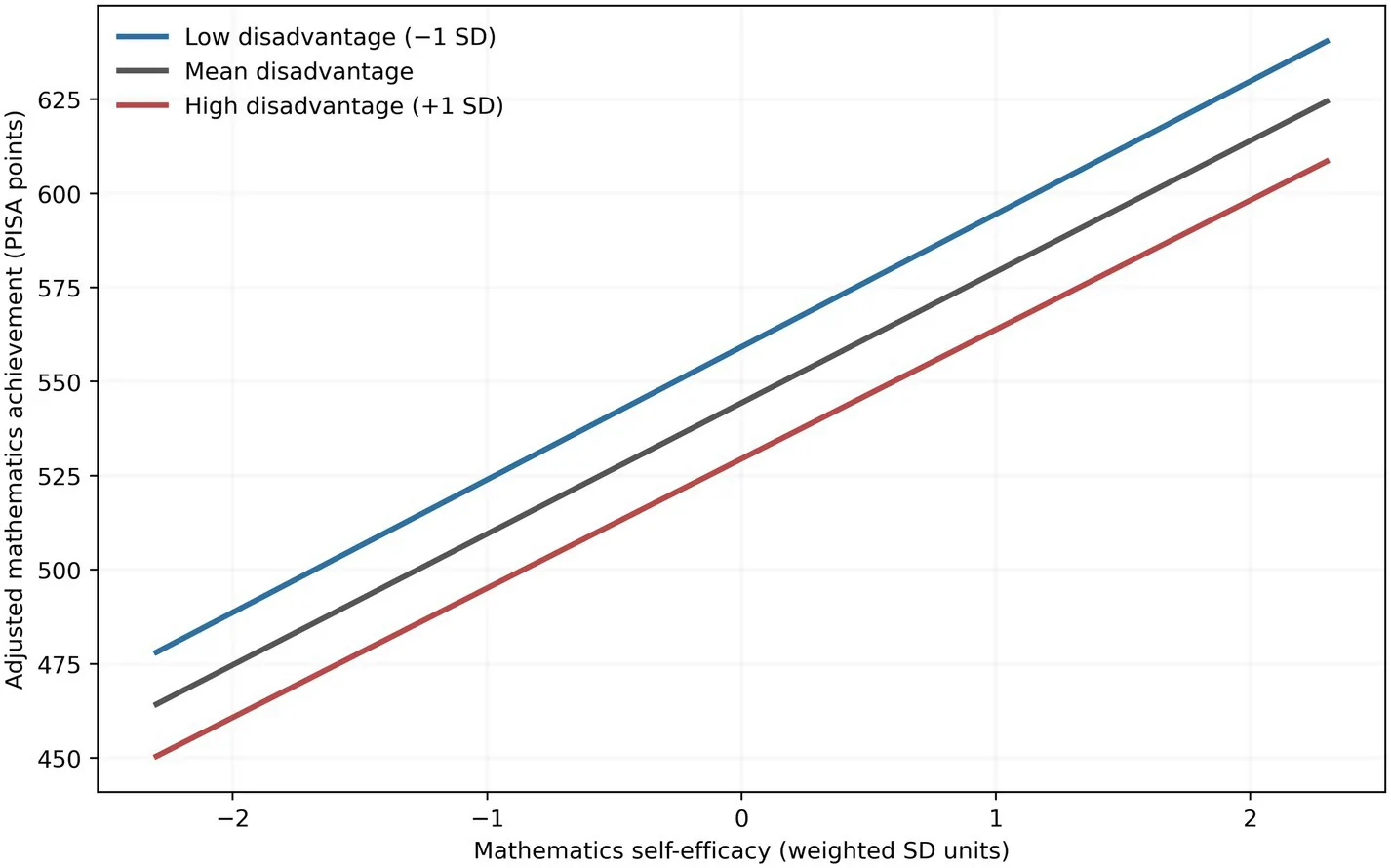
Adjusted mathematics achievement across mathematics self-efficacy at low, mean, and high socioeconomic disadvantage. The Fay-BRR pooled self-efficacy-by-disadvantage interaction was not statistically significant (*p* = 0.744).

**Table 4:** Simple slopes of mathematics anxiety and mathematics self-efficacy across socioeconomic disadvantage.

Predictor	Disadvantage level	*B*	SE	95% CI	*p*
Mathematics anxiety	Low (−1 SD)	−7.26	2.33	[−11.82, −2.70]	0.002
Mathematics anxiety	Mean	−9.03	1.53	[−12.02, −6.04]	<0.001
Mathematics anxiety	High (+1 SD)	−10.80	1.96	[−14.64, −6.96]	<0.001
Mathematics self-efficacy	Low (−1 SD)	35.28	2.47	[30.43, 40.13]	<0.001
Mathematics self-efficacy	Mean	34.82	1.62	[31.63, 38.00]	<0.001
Mathematics self-efficacy	High (+1 SD)	34.36	1.79	[30.86, 37.85]	<0.001

### 4.4 Robustness analyses

The bottom-SES-quartile sensitivity analysis led to the same substantive conclusion under Fay-BRR inference. Students in the bottom weighted ESCS quartile within their jurisdiction had an adjusted achievement difference of −21.90 points relative to the remaining 75%, SE = 3.71, 95% CI [−29.16, −14.63], *p* < 0.001. Mathematics anxiety remained negatively associated with achievement, *B* = −8.22, SE = 1.69, *p* < 0.001, and the anxiety-by-bottom-quartile interaction was not significant, *B* = −2.73, SE = 2.97, *p* = 0.357. Self-efficacy remained strongly positive, *B* = 36.71, SE = 2.10, *p* < 0.001, while the self-efficacy-by-bottom-quartile interaction remained non-significant, *B* = −1.51, SE = 3.05, *p* = 0.620 (Table 5).

**Table 5:** Bottom-SES-quartile sensitivity model.

Term	*B*	SE	95% CI	*p*
Mathematics anxiety (z)	−8.22	1.69	[−11.53, −4.92]	<0.001
Mathematics self-efficacy (z)	36.71	2.10	[32.59, 40.83]	<0.001
Bottom weighted ESCS quartile	−21.90	3.71	[−29.16, −14.63]	<0.001
Anxiety × bottom quartile	−2.73	2.97	[−8.55, 3.09]	0.357
Self-efficacy × bottom quartile	−1.51	3.05	[−7.48, 4.46]	0.620

Jurisdiction-specific Fay-BRR models also produced the same substantive pattern. In Hong Kong, anxiety was negative (*B* = −8.95, SE = 1.67, *p* < 0.001), self-efficacy was positive (*B* = 34.64, SE = 1.77, *p* < 0.001), and disadvantage was negative (*B* = −15.28, SE = 1.85, *p* < 0.001); neither interaction was supported (anxiety by disadvantage: *B* = −1.98, SE = 1.64, *p* = 0.229; self-efficacy by disadvantage: *B* = −0.45, SE = 1.56, *p* = 0.773). In Macao, anxiety was negative (*B* = −10.27, SE = 1.43, *p* < 0.001), self-efficacy was positive (*B* = 35.61, SE = 1.50, *p* < 0.001), and disadvantage was negative (*B* = −8.70, SE = 1.43, *p* < 0.001). The anxiety interaction was non-significant (*B* = −0.17, SE = 1.59, *p* = 0.914), while the self-efficacy interaction was *B* = −2.74, SE = 1.43, *p* = 0.055. Predictors were standardized using the pooled analytic sample, placing the jurisdiction-specific coefficients on a common predictor metric. However, the separately estimated jurisdiction models do not constitute formal tests of cross-jurisdiction coefficient equality; such differences would require explicit jurisdiction-by-predictor interaction tests or an equivalent joint-model test. Accordingly, these coefficients are presented as jurisdiction-specific sensitivity estimates. Table 6 summarizes the jurisdiction-specific focal coefficients.

**Table 6:** Jurisdiction-specific focal coefficients from continuous moderation models.

Jurisdiction	Term	*B*	SE	*p*
Hong Kong	Anxiety	−8.95	1.67	<0.001
Hong Kong	Self-efficacy	34.64	1.77	<0.001
Hong Kong	Disadvantage	−15.28	1.85	<0.001
Hong Kong	Anxiety × disadvantage	−1.98	1.64	0.229
Hong Kong	Self-efficacy × disadvantage	−0.45	1.56	0.773
Macao	Anxiety	−10.27	1.43	< 0.001
Macao	Self-efficacy	35.61	1.50	< 0.001
Macao	Disadvantage	−8.70	1.43	< 0.001
Macao	Anxiety × disadvantage	−0.17	1.59	0.914
Macao	Self-efficacy × disadvantage	−2.74	1.43	0.055

The pooled target represented by the final student weights is dominated by Hong Kong because the analytic-sample weight sum was 44,498.97 for Hong Kong and 4,344.82 for Macao, corresponding to 91.10 and 8.90% of the pooled weighted population. To test whether this composition drove the results, the full and replicate weights were rescaled to equal totals within the two jurisdictions. Under equal-jurisdiction weighting, anxiety remained negative (*B* = −9.50, SE = 1.11, *p* < 0.001), self-efficacy was positive (*B* = 35.41, SE = 1.17, *p* < 0.001), and disadvantage was negative (*B* = −12.71, SE = 1.05, *p* < 0.001). Neither interaction was supported (anxiety by disadvantage: *B* = −0.88, SE = 1.15, *p* = 0.441; self-efficacy by disadvantage: *B* = −0.87, SE = 0.99, *p* = 0.380) (Table 7 summarizes this weighting sensitivity).

**Table 7:** Jurisdiction weight composition and equal-jurisdiction sensitivity.

Quantity	Primary pooled weighting	Equal-jurisdiction sensitivity
Analytic n (HKG/MAC)	5,454/4,306	same students
Weight sum (HKG/MAC)	44,498.97/4,344.82	5,000/5,000
Weight share (HKG/MAC)	91.10%/8.90%	50.00%/50.00%
Anxiety B (SE)	−9.03 (1.53)	−9.50 (1.11)
Self-efficacy B (SE)	34.82 (1.62)	35.41 (1.17)
Disadvantage B (SE)	−14.88 (1.69)	−12.71 (1.05)
Anxiety × disadvantage B (SE)	−1.77 (1.52)	−0.88 (1.15)
Self-efficacy × disadvantage B (SE)	−0.46 (1.42)	−0.87 (0.99)

Complete-case screening excluded 531 of 10,291 students (5.16%), but the rate differed by jurisdiction: 453 of 5,907 Hong Kong students (7.67%) and 78 of 4,384 Macao students (1.78%). Excluded students had a lower PV-pooled weighted mathematics mean than retained students (503.47 vs. 544.29), a 40.81-point difference, and were more often male in the pooled sample (64.35% vs. 52.13%). Age differences were small, while the relative-grade difference was modest. Because this pattern indicates observable selectivity, the inverse-probability-of-complete-case sensitivity analysis was used. Its focal estimates remained close to the primary Fay-BRR results: anxiety *B* = −8.95 (SE = 1.57), self-efficacy *B* = 35.04 (SE = 1.65), disadvantage *B* = −14.84 (SE = 1.72), anxiety by disadvantage *B* = −1.84 (SE = 1.56, *p* = 0.239), and self-efficacy by disadvantage *B* = −0.46 (SE = 1.43, *p* = 0.748). Thus, adjustment for observed predictors of complete-case retention did not change the moderation conclusion, although unobserved causes of missingness cannot be ruled out. Table 8 reports the attrition comparison and inverse-probability sensitivity estimates.

**Table 8:** Complete-case attrition and inverse-probability sensitivity.

Quantity	Retained/primary	Excluded/IPCW sensitivity
Overall n	9,760	531
Hong Kong n	5,454	453
Macao n	4,306	78
PV-pooled mathematics mean	544.29	503.47
Weighted male percentage	52.13%	64.35%
Weighted age mean	15.81	15.82
Weighted grade mean	9.59	9.49
Anxiety B (SE)	−9.03 (1.53)	−8.95 (1.57)
Self-efficacy B (SE)	34.82 (1.62)	35.04 (1.65)
Disadvantage B (SE)	−14.88 (1.69)	−14.84 (1.72)
Anxiety × disadvantage B (SE)	−1.77 (1.52)	−1.84 (1.56)
Self-efficacy × disadvantage B (SE)	−0.46 (1.42)	−0.46 (1.43)

## 5 Discussion

The results provide a qualified answer to the central empirical question. Mathematics anxiety, mathematics self-efficacy, and socioeconomic disadvantage each showed independent conditional associations with mathematics achievement, whereas the two targeted interaction terms were not statistically supported under official Fay-BRR variance estimation. The equivalence sensitivity analysis further indicates that interaction magnitudes of at least five PISA points per one-SD-by-one-SD change are inconsistent with the observed data, although smaller interactions remain possible. Conclusions are therefore restricted to the two specified interactions, the explicitly stated ±5-point sensitivity margin, and the 2022 cross-sectional setting.

The negative anxiety coefficient is consistent with the broader evidence base, but the present multivariable estimate adds an important qualification. Meta-analytic studies have consistently reported an inverse mathematics anxiety-achievement relationship (Barroso et al., 2021; Namkung et al., 2019; Zhang et al., 2019), while Carey et al. (2016) and Ramirez et al. (2018) emphasize mechanisms such as worry, avoidance, attentional capture, and working-memory interference. In the present model, the anxiety coefficient is smaller than the zero-order association because mathematics self-efficacy, socioeconomic disadvantage, and the specified controls are considered simultaneously. This attenuation is theoretically informative rather than contradictory; it indicates that part of the bivariate anxiety-achievement relationship overlaps with other psychological and socioeconomic characteristics. The remaining coefficient, therefore, represents a conditional association rather than a universal effect size. The result is also compatible with prior evidence that the magnitude of the anxiety-performance relationship varies across measurement conditions, tasks, ages, and cultural settings (Namkung et al., 2019; Zhang et al., 2019).

Mathematics self-efficacy showed the largest focal coefficient, consistent with social cognitive accounts of perceived capability and persistence. The positive conditional association aligns with the long-standing evidence linking self-efficacy with academic performance and mathematical problem solving (Multon et al., 1991; Pajares and Miller, 1994), as well as with Usher and Pajares (2008) account of mastery experience, social persuasion, vicarious experience, and affective states as sources of efficacy beliefs. Importantly, the present finding does not establish a one-way causal pathway. Honicke and Broadbent (2016) identified limitations in causal evidence, and Talsma et al. (2018) found reciprocal relationships in which earlier performance can also shape later self-efficacy. Accordingly, the relatively large MATHEFF coefficient is best interpreted as a strong concurrent association between mathematics-specific perceived capability and achievement after adjustment for anxiety, disadvantage, and the specified controls. Its persistence across socioeconomic positions suggests that the association is not confined to a single level of disadvantage, although the non-significant interaction cannot establish exact equality of the slopes.

The independent association of socioeconomic disadvantage is also substantively important and places the psychological findings within a structural context. The negative disadvantage coefficient aligns with the broader literature showing persistent socioeconomic gradients in academic achievement (Bradley and Corwyn, 2002; Sirin, 2005; Berkowitz et al., 2017) and with Liu et al. (2020) synthesis of evidence from China. The bottom-ESCS-quartile sensitivity model strengthens the substantive interpretation by showing an adjusted difference of about 22 mathematics points for students in the lowest within-jurisdiction socioeconomic quartile. This pattern indicates that the achievement gradient remains visible even when mathematics anxiety and mathematics self-efficacy are included jointly. It therefore supports treating socioeconomic position as a substantive correlate of achievement rather than merely a background control. At the same time, the result should not be interpreted as identifying which component of ESCS—family resources, educational opportunities, or school-related conditions—produces the observed difference, because the present cross-sectional model does not isolate those mechanisms.

The moderation results refine the theoretical framework rather than overturn the established main associations. The resource-constraint argument proposed that limited compensatory support could make mathematics anxiety more consequential under disadvantage, whereas the resource-translation account suggested that limited resources could reduce the achievement return associated with self-efficacy. Neither interaction received statistical support in the present models. This contrasts with the broader proposition that motivational or psychological characteristics can condition socioeconomic achievement differences, as illustrated by Claro et al. (2016), but it is not necessarily inconsistent with that literature because the focal construct, outcome, population, and interaction tested here differ. Likewise, prior work shows that the anxiety-performance association is heterogeneous across contexts (Namkung et al., 2019; Zhang et al., 2019), yet such heterogeneity does not require ESCS to be the moderator responsible for that variation. The present evidence, therefore, narrows the claim to these two specified interactions in Hong Kong and Macao during the PISA 2022 cycle.

A second interpretation concerns statistical precision. Interaction effects are generally more difficult to estimate precisely than main associations, which is why both null-hypothesis and equivalence evidence were examined. The Fay-BRR 95% confidence intervals crossed zero for both interactions, so H4 and H5 were not supported. Using the explicitly stated +/−5-point sensitivity margin (approximately 0.05 of the pooled mathematics SD), however, the corresponding 90% confidence intervals fell entirely within the equivalence bounds. This supports practical equivalence only relative to that deliberately small sensitivity margin; it does not establish that the true interactions are exactly zero or that five points is a universal educational importance threshold.

The findings also address a common conceptual error: structural and psychological explanations need not be treated as mutually exclusive. The strong self-efficacy coefficient does not imply that socioeconomic inequality is unimportant, nor does the disadvantage coefficient imply that students’ beliefs and emotions are secondary. The fitted model displays these gradients simultaneously. The equal-jurisdiction and inverse-probability sensitivity analyses produced the same moderation conclusion, indicating that the result is not an artifact of Hong Kong’s larger weighted contribution or of the observed predictors of complete-case retention.

The non-significant interaction tests alone do not establish that the psychological slopes are identical across socioeconomic positions. However, the supplementary equivalence analysis using the explicitly stated ±5-PISA-point margin placed both 90% confidence intervals within the equivalence bounds. Accordingly, the present data support practical equivalence only relative to this sensitivity margin, while smaller interaction effects remain possible. Disadvantage was independently associated with mathematics achievement, but the analyses did not provide statistically reliable evidence that it moderated either psychological slope. Control-value theory remains useful for interpreting the negative anxiety-achievement association, while social cognitive theory remains useful for interpreting the positive self-efficacy-achievement association.

Methodologically, the analysis preserves all 10 mathematics plausible values and uses the 80 official PISA Fay-BRR replicate weights for design-based sampling variance. Mathematics descriptives and achievement correlations were calculated separately for each plausible value and then pooled, avoiding student-level averaging of the 10 plausible values for inferential summaries. Equal-jurisdiction weighting, bottom-quartile coding, jurisdiction-specific estimation, and inverse-probability-of-complete-case weighting were employed to examine whether the focal conclusions depended on population weighting, moderator coding, jurisdiction pooling, or observed complete-case selectivity.

The findings call into question the singular approach to intervention, both for the school and for policy. Anxiety reduction in mathematics teaching, low threat assessment of mathematics, opportunities for error correction, and explicit strategies to help students cope with evaluative stress could be important not just for disadvantaged students, but generally. Instructional strategies that foster real-life experiences of success, provide calibrated feedback, and make difficulty seem manageable may also be broadly applicable, as self-efficacy is linked to achievement across the same range. The recommendations are similar to those presented in the literature on mathematics anxiety and self-efficacy, and the present cross-sectional analysis does not assess the effects of interventions.

A parallel structural response is necessary regarding the disadvantage gradient. The psychological variables were controlled, and lower achievement was associated with lower ESCS. That trend aligns with policies that provide increased access to quality instructional content, academic and other support, technology, enrichment, and protected learning time. The null interactions do not detract from the case for these policies. Rather, they suggest that socioeconomic support should not be viewed merely as a means to reduce the detrimental effects of anxiety and to enhance the productive effects of self-efficacy. This is important because the achievement gradient is a reality in itself.

For Hong Kong and Macao, interpretation should remain jurisdiction-specific rather than being extended to a national Chinese adolescent population. The two systems are analyzed together only after including jurisdiction in the pooled model, and jurisdiction-specific models are also reported to show whether the substantive pattern is driven by one system. The practical implication is therefore local and conditional: students showing high mathematics anxiety may warrant lower-threat assessment practices, structured opportunities to correct errors, and access to counseling or learning support; students with weaker mathematics self-efficacy may benefit from appropriately challenging tasks, mastery-oriented feedback, and repeated opportunities to demonstrate competence. Socioeconomically disadvantaged students may additionally require access to instructional materials, tutoring, digital resources, enrichment opportunities, and protected study time. These recommendations are consistent with the observed associations but do not constitute tested intervention effects, and differences in the educational contexts of Hong Kong and Macao should not be conflated into claims about China.

These are both indicative of an educational institution, not determinative. Psychological and instructional support for a student exhibiting high anxiety and low self-efficacy is warranted, irrespective of socio-economic status. Anxiety may be low and self-efficacy may be high, but a student in a socio-economically disadvantaged group can still benefit from resource support. Considering these factors in isolation can lead to schools assuming that a positive factor will offset the impact of other negative factors. The findings thus indicate that a hierarchical approach to supporting all barriers to learning—emotional, motivational, and material—is the most appropriate model.

## 6 Conclusion

This study examined whether socioeconomic disadvantage moderates the associations of mathematics anxiety and mathematics-specific academic self-efficacy with mathematics achievement among adolescents in Hong Kong (China) and Macao (China). In the analyzed PISA 2022 sample, greater mathematics anxiety was associated with lower mathematics achievement, stronger mathematics-specific self-efficacy was associated with higher achievement, and greater socioeconomic disadvantage was associated with lower achievement after adjustment for the specified controls. The hypothesized anxiety-by-disadvantage and self-efficacy-by-disadvantage interactions were not statistically supported.

These findings indicate that emotional, motivational, and socioeconomic conditions are concurrently related to mathematics achievement, while the present analyses provide insufficient evidence that socioeconomic disadvantage reliably alters either psychological-achievement association. The non-significant interaction estimates alone do not demonstrate that the true interactions are zero or that the conditional slopes are exactly equal. However, the supplementary equivalence analysis supports practical equivalence relative to the prespecified sensitivity margin of ±5 PISA points, while smaller interaction effects remain possible. Accordingly, conclusions are limited to the tested interaction specifications, this stated equivalence margin, and the cross-sectional associations observed in the two jurisdictions.

The study contributes by examining mathematics anxiety, mathematics-specific academic self-efficacy, and socioeconomic disadvantage within a single moderation framework while retaining all 10 mathematics plausible values. Substantively, the results support considering psychological and socioeconomic barriers together rather than treating either as a substitute for the other. Schools may therefore consider support for mathematics-related anxiety and task-specific efficacy alongside measures that improve access to learning resources and academic support, while recognizing that the present observational design does not establish intervention effects.

Interpretation remains bounded by the cross-sectional design, the geographic scope of Hong Kong and Macao, the mathematics-specific measures, and item-level missingness. Official Fay-BRR replicate weights and all 10 mathematics plausible values are incorporated in the primary inference, but weighting cannot eliminate every source of bias. Hong Kong’s PISA 2022 student response rate was 75%, and school response rates were 60% before and 80% after replacement, below the relevant PISA targets; residual unit non-response bias therefore remains possible. This survey-level unit non-response is distinct from the additional 5.16% complete-case attrition. Observable attrition was assessed, and inverse-probability weighting produced similar focal estimates, but unobserved selection cannot be excluded. Longitudinal evidence is still required to establish temporal ordering among achievement, anxiety, and self-efficacy.

## Data Availability

Publicly available datasets were analyzed in this study. This data can be found at: https://www.oecd.org/en/data/datasets/pisa-2022-database.html and https://www.oecd.org/pisa/data/2022database/.
